# Medication-Related Osteonecrosis of the Jaw: A Critical Narrative Review

**DOI:** 10.3390/jcm10194367

**Published:** 2021-09-24

**Authors:** Alejandro I. Lorenzo-Pouso, José Bagán, Leticia Bagán, Pilar Gándara-Vila, Cintia M. Chamorro-Petronacci, Pablo Castelo-Baz, Andrés Blanco-Carrión, María Ángeles Blanco-Fernández, Óscar Álvarez-Calderón, Javier Carballo, Mario Pérez-Sayáns

**Affiliations:** 1 Oral Medicine, Oral Surgery and Implantology Unit (MedOralRes), Faculty of Medicine and Dentistry, Universidade de Santiago de Compostela, 15782 Santiago de Compostela, Spain; pilar.gandara@usc.es (P.G.-V.); cintiamica.chamo@yahoo.es (C.M.C.-P.); andres.blanco@usc.es (A.B.-C.); mario.perez@usc.es (M.P.-S.); 2 Department of Stomatology and Maxillofacial Surgery, Hospital General Universitario de Valencia, 46014 Valencia, Spain; jose.v.bagan@uv.es (J.B.); leticia.bagan@uv.es (L.B.); 3 ORALRES Group, Health Research Institute of Santiago de Compostela (FIDIS), 15782 Santiago de Compostela, Spain; 4 Deparment of Surgery and Medical-Surgical Specialties, Faculty of Medicine and Dentistry, Universidade de Santiago de Compostela, 15782 Santiago de Compostela, Spain; pablo.castelo.baz@usc.es; 5 Stomatology Service, Provincial Hospital of Pontevedra, 36002 Pontevedra, Spain; mbfdez10@hotmail.com; 6 Department of Health Sciences, University of Coruna, 15001 La Coruña, Spain; oscaraci.udc@gmail.com; 7 Department of Food Technology, Faculty of Sciences, University of Vigo—Ourense Campus, 32004 Ourense, Spain; carbatec@uvigo.es

**Keywords:** jaw osteonecrosis, medication-related osteonecrosis of the jaw, osteonecrosis of the jaw, wound healing

## Abstract

Background: Nearly two decades have passed since a paradoxical reaction in the orofacial region to some bone modifying agents and other drugs was recognized, namely medication-related osteonecrosis of the jaw (MRONJ). Purpose: The aim of this manuscript was to critically review published data on MRONJ to provide an update on key terminology, concepts, and current trends in terms of prevention and diagnosis. In addition, our objective was to examine and evaluate the therapeutic options available for MRONJ. Methods: The authors perused the most relevant literature relating to MRONJ through a search in textbooks and published articles included in several databases for the years 2003–2021. Results and conclusions: A comprehensive update of the current understanding of these matters was elaborated, addressing these topics and identifying relevant gaps of knowledge. This review describes our updated view of the previous thematic blocks, highlights our current clinical directions, and emphasizes controversial aspects and barriers that may lead to extending the accumulating body of evidence related to this severe treatment sequela.

## 1. Introduction

Avascular necrosis, also known as osteonecrosis, is a generic term referring to the ischemic death of the constituents of bone [1,2]. Osteonecrosis has a wide variety of causes and can affect nearly any bone in the human body, counterpart individually each osteonecrosis-affected bone has unique clinical, etiologic, and prognostic factors. Bone infarction begins when the blood supply is discontinued. Once this infarct is established, a central necrotic core surrounded by an ischemic zone is commonly found [3,4]. Osteomyelitis of the jaw is a kind of avascular necrosis, characterized by infection and inflammation of the bone marrow in the bones of the jaw (i.e., maxilla and/or the mandible) [5,6]. In contrast to avascular osteonecrosis in other bones, in jaws, the main cause is the spread of adjacent odontogenic infection, whereas the second most common cause is trauma. During the 19th and early 20th centuries, two historic occupational diseases brought on by the ingestion and subsequent absorption of radium and white phosphorus into the bones were described [7,8]. A related condition, initially named bisphosphonate-associated osteonecrosis of the jaw (BRONJ), was initially described as a side-effect of amino-bisphosphonates by Marx in 2003 [6], a class of phosphorus-based drugs that inhibit bone resorption which were and are used widely for treating osteoporosis, bone metastases in cancer and some other conditions mainly to reduce fractures incidence fractures and other skeletal-related events [9].

Five years later, new oncology drugs and other types of antiresorptive therapies were progressively linked to this avascular necrosis from case reports to adverse events of long-term follow-up of randomized trials [10,11]. In addition, anti-angiogenic agents such as monoclonal antibodies against VEGF (i.e., Bevacizumab, Aflibercept), tyrosine kinase inhibitors (i.e., Sunitinib, Sorafenib, Imatinib, Axitinib, and Cabozantinib), and mTOR inhibitors (i.e., Temsirolimus and Everolimus) [12,13,14]. Although, some of these cases are related to a concomitant consumption of antiresorptive drugs or to a previous discontinuation [12,15]. Other drugs were also related to this type of jaw avascular necrosis such as anti-TNF factors (Etanercept, Adalimumab, Infliximab), anti-CD20 antibodies (Rituximab), and other immunosuppressive drugs such as methotrexate, prednisolone, or tocilizumab. Even some selective estrogen receptor modulator such as tamoxifen and raloxifene has been related to MRONJ [13,16]. The list of agents responsible for this outcome continues to rise but a poor level of evidence is generalized due to this level being based mainly on case reports and case series which jeopardize the current understanding of this adverse event [17]. Moreover, it is important to remember that exposed bone or sequestra can occur in patients not exposed to these drugs [18,19].

The explained rationale switched the definition to medication-related osteonecrosis of the jaw (MRONJ). This term is supported by the American Association of Oral and Maxillofacial Surgeons (AAOMS) in its latest position paper update [20]; although it is worth mentioning that the 2015 International Taskforce on Osteonecrosis of the Jaw consensus paper removed anti-angiogenics due to a lack of evidence at the time [21]. Two previous position papers, one by the American Society for Bone and Mineral Research (ASBMR) back in 2007 and another previous one by the AAOMS started touting this treatment sequela [20,22]. So was conceived a severe bone disease currently known as MRONJ.

The objectives of this study were: to critically review published data on MRONJ to provide an update on key terminology and concepts and current trends in terms of prevention and diagnosis. Finally, we evaluate the possible usefulness of current treatment strategies in MRONJ.

## 2. Methods

All papers and clinical reviews of MRONJ in the electronic databases (Medline, Embase, Scopus, and the Cochrane Library) published from January 2003 to January 2021 in any language have been evaluated. We also perused relevant textbooks and abstracts from our institution’s library catalog. Independent research in relevant related-content journals was also performed, including Bone; British Journal of Oral and Maxillofacial Surgery; International Journal of Oral and Maxillofacial Surgery; Journal of Bone and Mineral Research; Journal of Oral Maxillofacial Surgery; Journal of Oral Pathology and Medicine; Journal of Cranio-Maxillofacial Surgery; Oral Diseases; Oral Oncology and Oral Surgery, Oral Medicine Oral Pathology, and Oral Radiology. We also examined the references of every article retrieved and those of recent reviews to trace further publications or reports.

## 3. Definition

AAOMS update of 2014, reported that a patient is considered to have MRONJ if all the following conditions are met: (i) current or past treatment with antiresorptive or antiangiogenic drugs; (ii) exposed bone or intra- or extraoral fistulisation in the maxillofacial region communicating with the bone and persisting for more than 8 weeks; (iii) no history of maxillary radiotherapy or clear maxillary metastatic disease [20].

It is worth mentioning that AAOMS and ASBMR definitions slightly differ. AAOMS introduces the statement “obvious metastatic disease”, which is not included in the article from the ASBMR position paper [20,21].

As stated, the diagnosis is essentially clinical-driven, although with nuances [23,24]. AAOMS did introduce a possible definition of this outcome (referred to as stage 0) as some authors suggested, corresponding to patients with symptoms but no exposed bone [25,26]. In this vein, AAOMS at the time of its latest position paper considered this variant as prodromal, and that over time up to 50% of these patients will progress to MRONJ stages 1, 2, or 3 [20]. In fact, later research confirmed this progression rate to frank bone exposure may be plausible [27]. Nonetheless, some authors considered this point-of-view erroneous due to it neglects of up to a quarter of affected individuals, so on being precipitant for delayed diagnosis and ultimately contributing to poorer control [28]. In this sense, this group of experts suggests that radiological examination via orthopantomography, cone-beam computed tomography, and magnetic resonance imaging are mandatory exploratory measures to complement clinical examination to detect plausible stage 0 cases but also on other stages to assess their extent and involvement of neighboring tissues [29]. AAOMS stated that this stage may overestimate the true disease frequency by including false-positive values due to its based on nonspecific clinical findings, radiographic changes, and symptoms that may overlap with other jawbone alterations such as osteomyelitis, osteoradionecrosis, alveolar osteitis, sinusitis, fibro-osseous lesions, chronic sclerosing osteomyelitis or oral ulceration and bone sequestration [20]. This ambiguity emphasizes the need for a more precise definition of non-exposed-MRONJ to address this complex differential diagnosis [30].

## 4. Pathogenesis

Despite the increasing amount of literature generated over the years, the pathogenesis of MRONJ is still not completely elucidated [31]. Many theories have been proposed for the pathogenesis of MRONJ [32]. Taken together, this research suggests it is probably multifactorial, with important roles for infection, inflammation, and trauma to the bone or soft tissue amplified by an altered bone remodeling or over suppression of bone resorption and angiogenesis inhibition [23,33,34]. Other alternative theories or others trying to integrate all these factors were also postulated or even partially studied [30]. These three main etiological fractions are discussed in-depth.

### 4.1. Infection, Inflammation, and Trauma

Invasive dental treatments (IDTs) and periodontal disease (PD) have been considered as potential risk factors of MRONJ; however, the association between these exposures and MRONJ remains controversial [35]. Dental treatments are considered IDTs when the treatments may cause bleeding and introduce oral bacteria into the bloodstream, such as extraction, scaling and root planning, implant placement, and any kind of oral surgery. IDTs as PD can yield temporary bacteraemia able to cause a microbial immune subversion that triggers systemic inflammation. In this vein, scanning electron microscope analysis from MRONJ lesions has revealed microbial biofilm formation on sequestered bone, and in this dysbiosis seems to be the regular presence of Actinomyces species [36]. The critical role of bacterial infection in the pathogenesis of MRONJ may be justified by its decreased incidence in patients following improvement in their dental hygiene [35,37,38]. Several animal models have shown that inflammation or bacterial infection and systemic antiresorptive drugs are sufficient to induce MRONJ and that at the same time the presence of previous necrosis seems not to be a prerequisite [39,40]. In this context, some of the bacteria responsible for this dysbiosis can produce lipopolysaccharides that are able to increase cytokine production or directly regulate the production of receptor activator of nuclear factor κB ligand (RANKL). These circumstances can modify the bone matrix by osteoclast reprogramming. These cells may produce an exacerbated number of osteolytic proteins such as H^+^-ATPases, V-ATPase, and chloride channel 7 [41,42]. This process alters natural homeostasis triggering acidification processes able to alter bone turnover [23]. In this vein, Otto et al. postulated that localized change in pH caused by dentoalveolar infections or IDTs may be the initial context of MRONJ onset [43]. This group later demonstrated in vitro that bisphosphonates and a local acidic milieu reduce cell viability and activity of immortalized mesenchymal stem cells [44]. Dayisoylu et al. also demonstrated that an alkaline environment can prevent MRONJ by an in vivo study [45]. It is also worth mentioning that recently indigenous microbiota seems to protect against MRONJ onset according to a mice-based study [46].

Apart from these mechanisms, altered host immune response sense another as important as the infection itself. The immune cells and macrophages are involved in the wound healing process [47]. It has been suggested that macrophages may initially bond to bisphosphonates instead of osteoclasts and the presence of this antiresorptive significantly alters macrophage viability and morphology in vitro [40,48]. This theory seems valid considering the lack of affinity between bisphosphonates and osteoclast and the superior accumulation of these drugs on jaws in relation to the rest of the skeleton [31].

### 4.2. Altered Bone Remodelling or Oversuppression of Bone Resorption

Bisphosphonates and other antiresorptive drugs, inhibit osteoclast differentiation and increase cell death. Moreover, adequate bone remodeling capacity is thought to be critical in the defense against infection and accumulating microfractures [49,50]. The increased bone resorption in the setting of oral conditions, coupled with the thin overlying mucosa and a direct pathway through the periodontal ligament with the external environment, make the jaws a suitable breeding ground for MRONJ to develop [51]. It is worth mentioning that BPs can play a pivotal role in mesenchymal stem cells of the oral cavity. Particularly, BPs at periodontal ligament stem cells can cause impairment by inducing apoptosis in a dose-dependent manner [52,53]. To combat the effects of bone turnover suppression at the jawbone, withdrawing antiresorptive medications before tooth extraction of surgical procedures is often advocated to potentially reduce the risk of MRONJ, and in this line, the concept “drug holidays” was made. No human studies have evidence regarding its usefulness [54,55]; nonetheless, an animal-based study showed this method as promising both in the case of denosumab and bisphosphonates [54]. Other alternatives to mitigate this impaired bone remodeling have been proposed such as the use of parathyroid hormone and its derivatives or optimal daily vitamin D intake [20].

### 4.3. Altered Angiogenesis

Bone becomes necrotic without adequate blood supply [56]. In the case of MRONJ, bisphosphonates can contribute to the pathogenesis of MRONJ due to their ability to reduced blood vessel formation causing delayed mucosal healing [57]. It has been reported that various antiangiogenic agents, such as VEGF antibodies and tyrosine kinase inhibitors, can also cause jaw necrosis and therefore the development of MRONJ [15]. Unfortunately, it is still unclear if and how simultaneous or time-shifted use of bisphosphonates and further antiangiogenic agents increases the risk and the extent of MRONJ development as debated by the AAOMS and ASBMR [20,21]. The bisphosphonate compound zoledronic acid indirectly impaired angiogenesis via targeting MMP9 expressing macrophage, which, curiously, is one of the most altered molecules in the pathogenesis of periodontal diseases. It is plausible that bisphosphonates can block angiogenesis by interfering with endothelial cell proliferation and survival via apoptosis although the level of evidence is low some studies at a genetic and transcriptional level have help for the field to move forward [48,58,59].

In the case of denosumab, this monoclonal antibody seems to not induce soft tissue toxicity so in this vein angiogenesis has not proved to be linked to this kind of lesions [60]. Nevertheless, patients who have taken bisphosphonate drugs at any time in the past and those who have taken denosumab in the last nine months are allocated to a risk group as if they were still taking the drug [61].

## 5. Incidence

Rare outcomes require an evidence-based estimate of the global point prevalence to inform public policy. Estimating the global prevalence of rare drug adverse events is challenging foremost for the diversity of the data, which are derived from a variety of disparate information sources that are not standardized and difficult to pool [62]. Anecdotally, a Swedish retrospective cohort study reported that a general dentist practitioner may expect to see a case of MRONJ every 62 years in clinical practice [63]. The risk of MRONJ among patients with cancer exposed to antiresorptive or antiangiogenic medication is about 1% (range 0.2–6.7%), whereas this risk is about 0.1% (range 0.004–0.2%) among patients who are being treated for osteoporosis using antiresorptive therapy. The risk of MRONJ is greater in patients with cancer than in those receiving antiresorptive treatments for osteoporosis by a factor of 10 [64]. A recent meta-analysis found that the use of denosumab is associated with a significantly higher risk of developing MRONJ compared to zoledronate in cancer patients, although authors reported serious plausible biases within its report [65]. A recent multicenter retrospective cohort study involving 22 secondary care centers elucidated data stratified by bisphosphonates type, a time of 6.0 and 2.2 years of oral alendronate and intravenous zoledronate therapy, respectively, and a time of 5.3 and 2.2 years of therapy is required for 50% of patients with osteoporosis and cancer to develop MRONJ [66]. To conclude, all these numbers are simply approximations, but they can be promptly translated into clinical application to inform the design of clinical trials, epidemiological studies. Further research, notably through long-term population registries and the implementation of a specific codification in healthcare systems, will help to refine our presented MRONJ estimates in terms of epidemiology [67].

## 6. Risk Factors

Triggering factors and risk factors for MRONJ, comorbidities, and medications should be explored as examined individually in light of the foregoing considerations.

Triggering factors risk factors, and so the first group, are classically identified as IDT or oral pathologies such as PD. The most reported dental risk factors are classically dental extraction, periodontal disease, other kinds of oral surgeries, dental implant placement, infection/abscess, or trauma derived from ineffective prosthetic solutions [10,35,68,69]. There has been clinical evidence that wearing ill-fitting dentures is also one of the MRONJ risk factors [70]. Nonurgent procedures should be delayed [21]. It is possible that some of the cases described can be linked mainly to just single local risk factors use but to rule out a putative risk factor based on the presence of another appears inappropriate [71]. Risk factors are not etiologic agents, and such an approach would not allow the identification of new risk factors or categorization of the present ones [72]. Therefore, it is reasonable to suggest that optimizing the health of the oral cavity by reducing inflammatory and infection burden to prevent the need for future invasive treatment must be the prudent strategy in at-risk patients [73]. It is worth mentioning that the only independent risk factor linked to MRONJ is tooth extraction despite the advanced identification of other risk factors [33]. Although, some controversies are still discussed due to some authors reporting that underlying pre-existing dental/periodontal infection rather than the surgery per se may act as the real starting point [74,75].

An interdisciplinary and oncologist, rheumatologist general dental professionals are essential [76]. A dentist should also be provided with information about the patient’s medical diagnosis and current therapy, or the future therapy established and its duration. Controllable risk factors (i.e., modifiable) should be minimized, and comprehensive oral care monitoring as recommended by the AAOMS should be established [20].

A recent systematic review pooled 39 different systemic diseases and 14 medical conditions as potential MRONJ risk factors [35]. In the case of systemic diseases, excluding those involving the use of these drugs, cardiovascular diseases and rheumatoid arthritis are highlighted [77,78,79]. Among the medical factors: chemotherapy, corticosteroids, smoking vitamin D deficiency, renal dialysis, anemia, Paget’s disease of bone, erythropoietin therapy, cyclophosphamide therapy, alcohol intake, and obesity are the most frequently reported [80,81,82]. Due to the scarce number of longitudinal studies involving this outcome, results make it difficult to quantitatively assess the thresholds for the level of damage of each of them individually [83].

It has also been well documented that advanced age is one of the significant risk factors for developing MRONJ. Occurring most commonly in sexagenarians and septuagenarians. Thus, in the day-to-day practice of gerodontology, strict surveillance is of paramount importance.

Whether genetic variation is a predictor for the development of medication-related osteonecrosis of the jaws (MRONJ) has been recently addressed in a systematic review finding that all studies have failed to show a single gene as a risk factor for MRONJ [84]. In terms of biomarkers at a translation or post-translational level according to recent reviews, none has achieved prominence or efficiency [85,86]. Marx et al. once identified serum CTX as a useful tool for risk assessment and treatment planning. This proposal has been strongly discarded by further studies [87]; all biomarker studies are invalid because all are made after the time-point of diagnosis, so the notion of prospection is totally absent [23]. Nowadays, it remains impossible to use any available biomarker with predictive or prognostic utility for these outcomes.

## 7. Prevention

Patients at higher risk for MRONJ should be placed on short follow-up intervals to maintain oral health and identify necrosis at the earliest stage possible. The onset of MRONJ may be subtle; so routine radiographic evaluation for hard tissue radiolucency may indicate the onset of an early stage (i.e., 0). So, in any kind of suspicious jaw region of at-risk patients, they are encouraged [25,27,28,29].

Several prophylactic protocols have been proposed for preventing this complication, including antiseptic rinses immediately before extraction and until healing of the socket, antibiotic prophylaxis, alveoloplasty with primary closure, fibrin, or autologous platelet-rich plasma, ozone therapy, limitation of the number of extractions performed in each session [69,76,88]. According to a recent metanalysis including six studies with 2332 cancer patients, dental preventive measures decreased MRONJ incidence by 77.3% (95% CI = 47.4–90.2%; *p* = 0.001) compared to at-risk control groups [69]. Particularly, the efficacy of autologous platelet concentrate (APC) applications in the prevention of MRONJ together with surgical debridement has not proved sufficient effectiveness for implementation [89].

In terms of non-dentoalveolar-related measures, the drug holiday concept (i.e., temporary discontinuation of the medication) in at-risk patients has been the subject of debate. Only one study to date confirmed this rationale [90] but based on the current body of evidence it seems negligible [55]. Theoretically, the risk of MRONJ diminishes when the frequency of administration of medications is reduced, so a reduced drug schedule may be a useful tool to prevent this severe adverse effect [91]. The half-life of bisphosphonates has been reported to be more than 10 years due to the higher affinity to hydroxyapatite, although that of denosumab is about 26 days after administration [92]. On the other hand, it is well known that suspension of drug therapy can create different cost–benefit balances, according also to the type of medication used; in some cases, even this drug suppression can affect the primary disease that precipitated the use of these drugs triggering an even worse outcome in the well-being of patients. It is important to reinforce the concept that the typical patient with MRONJ, apart from it, generally suffers general frailty. So, each case must be individualized for the benefit of the patient bearing in mind the entire conundrum of pathologies [93]. Taking together available recommendations on the management of individuals using or scheduled for their intake are hindered by controversy and a lack of evidence, and merely a reflection of panels of expert opinions [94,95].

## 8. Staging and Prognostication

**Figure 1:**
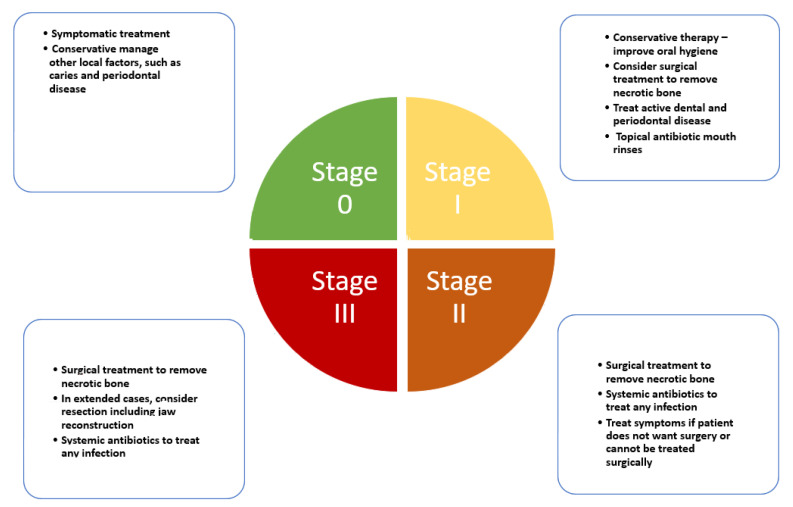
Clinical pathway for medication-related osteonecrosis of the jaw according to AAOMS latest update.

One of the most important issues in the development of a staging system for MRONJ is to aid in the selection of appropriate treatment and foresee a prognosis. The establishment of globally accepted criteria for the management of patients is imperative to achieve proper treatment approaches. However, from the introduction of the initial introduction by Ruggiero et al. [96], new staging systems have been constantly introduced jeopardizing the body of literature produced year after year in terms of longitudinal studies [97]. In this section, we report and discuss the most current accepted and up-to-date staging system to achieve consensus [20] (Figure 1). Therefore, current treatment strategies for MRONJ have been constructed based on clinical aspects rather than scientific evidence. The treatment strategy for MRONJ at each stage is introduced based on several position papers, and other studies including systematic reviews and consensus statements [20,21,23,30,82].

The following contents are an elaboration of how such staging is designed to combat MRONJ stages and to improve understanding and individual decision-making.

### 8.1. Stage 0

Stage 0 is characterized as a non-exposed variant of MRONJ and presents with nonspecific clinical signs, symptoms, and radiographic features such as: obscuring of the periodontal ligament, changes to the trabecular pattern, or osteosclerosis [98,99]. Reports have shown that the so-called nonexposed form of MRONJ might represent 13 to 20% of all cases of MRONJ [100]. It has been reported that 50% of patients in Stage 0 have progressed to a worse staging [101]. It is worth mentioning that diagnosing patients who have some symptoms without exposed bone as Stage 0 MRONJ, may also produce overdiagnosis [102]. For this rationale, specifical diagnostic tools are necessary [29]. However, symptomatic treatment and conservative management are recommended for patients with Stage 0 [25].

### 8.2. Stage 1

AAOMS defined this stage as a clinical scenario where exposed and necrotic bone, or fistulae that probes to be bone coexists in patients who are normally asymptomatic and have no evidence of infection [20]. Less frequently other oral signs are displayed such as: dental mobility; mucosal fistula; swelling; abscess; trismus; mandibular deformity or local hypoesthesia [103]. It may also present with radiographic findings overlapped with those described in Stage 0 [104,105].

### 8.3. Stage 2

This stage is characterized by exposed and necrotic bone or fistulae that probe to bone, with evidence of infection. These patients are typically symptomatic. These patients may also present with radiographic concordant with Stage 0.

### 8.4. Stage 3

Clinical signs and symptoms are the same as stage 2. Nonetheless, according to AAOMS one of the following features must also be present to establish diagnosis: exposed necrotic bone extending beyond the region of alveolar bone, i.e., inferior border and ramus; jaw fracture; extra-oral fistula; oral antral/oral–nasal communication; osteolysis extending to the inferior border of the mandible or sinus floor [20]. Some radiographic signs are also characteristic, such as diffuse osteosclerosis, with or without the following signs combined with the prominence of the inferior alveolar nerve canal, periosteal reaction, or sequestra formation.

Any classification should include all clinical possibilities to render studies comparable. The latest AAOMS 2014 update seemed to adopt all the proposals and critics. In this vein, we considered it as a quasi-gold standard able to guide MRONJ research properly [20]. Nevertheless, in our modest opinion, there are a few prevailing concerns about this classification: (i) MRONJ Stages 2 and 3 definitions are difficult to distinguish clearly, since some position papers, clinical reviews, and a clinical guideline do not provide the exact limit to establish exact thresholds [106]; (ii) the importance of the presence or absence of symptoms (i.e., divide each phase into a or a b according to presence or absence [97]; (iii) present classification/staging does not adequately capture the extension and severity of bone affected [30].

## 9. Management

There is no gold standard therapy defined in the literature, and the successful treatment of MRONJ remains elusive [3]. Expert opinion-based recommendations for the management of MRONJ included in the latest AAOMS position paper are primarily based on staging [20]. The goal of MRONJ therapy should be control of infection, progression of bone necrosis, ease associated pain, and ultimately improving the quality of life of patients [107].

According to the AAOMS report, the treatment modality for the initial stages should always be conservative and elective surgical procedures should be avoided [20]. Added to staging, conservative shall be initiated based on an in-depth microbiological study (i.e., bacterial cultivation and susceptibility testing) and radiological analysis (i.e., CT, MRI, or nuclear imaging) [108,109].

Commonly establish a treatment for 10–15 days with the appropriate antibiotic, in parallel with chlorhexidine rinses (once every 12 h for a month). In the case of normal flora, it is recommended to use amoxicillin/clavulanic acid, clindamycin, although the use of tetracyclines is also accepted [110]. In the case of the presence of bacterial species resistant against β-lactamase inhibitors, fluoroquinolones are the ones to use [111]. The healthcare professional should carry out irrigation of the exposed necrotic bed with 0.12% chlorhexidine—once every 72 h for four weeks. Then, the lesion should be re-evaluated one month later. If an improvement is confirmed the patient should continue with the 0.12% chlorhexidine rinses for another month and the professional’s application every 72 h [111,112]. In the case of a continuum suppuration, a curettage and regularization of the areas with irregular bone anatomy is recommended. In those cases, showing small areas of bone exposure after the first revaluation, the use of a soft laser to treat the necrotic bone to reach the bleeding healthy bone is recommended [113]. This minimally invasive technique allows to create micro-perforations on the bone basis and thus stimulating angiogenesis and mitigating inflammation and infection consequently [114].

Such planning is recommended in cases where there is no obvious disease progression, or uncontrolled pain because of MRONJ. It is essential to consider the individual response to treatment to switch to surgical approaches. In this vein, our opinion differs from the AAOMS mantra that the treatment modality should always be conservative and elective surgical procedures should be avoided due to a risk of extension of the areas of bone exposure or aggravation of the symptoms [115]. As related at a Workshop of the European task force on medication-related osteonecrosis of the jaw position document, a recent body of evidence has evidenced the equal or even better performance of early surgical interventions versus conservative approaches in early stages of MRONJ [30,104,116]. It is also worth mentioning that to surgically treat patients in a bad condition or with a poor life expectancy is not reasonable [33].

Surgery approaches are therefore indicated for patients with MRONJ whose disease does not respond to conservative treatment or is deemed unlikely to respond to conservative approaches from the start due to its advanced stage [117]. We believe that the protocol described by Wilde et al. is the most accurate in terms of our department’s results: “A full-thickness mucoperiosteal flap should be high and extended to reveal the entire area of exposed bone and beyond to disease-free margins; resection of the affected bone should be extended horizontally and inferiorly to reach healthy-appearing, bleeding bone; sharp edges should be smoothed; and primary soft tissue closure achievement” [118]. We defend the use of platelet-rich concentrates, preferable leukocyte platelet-rich fibrin, following Choukroun’s method but always associated with surgical management [119,120]. Although, it is worth mentioning that according to a recent systematic review the application of APCs did not show an unequivocal improvement versus surgical treatment alone [89].

According to a recent systematic review, a diversity of promising flap options was reported by several authors with promising results (for an extended overview please see [121]). Particularly our group, in cases of advanced MRONJ stages, opts to perform the bone resection and, when necessary, reconstruction of the area with a microvascularized flap [122]. We also reported that surgical neurolysis of the inferior alveolar nerve may be considered as the choice therapeutic technique to treat neuropathic pain when presented after initial surgery [123]. It is worth mentioning that the use of obturators of MRONJ cases allocated at the upper jaw has been also certified as a promising intervention [124]. There are also some pioneering experiences performed by a limited group of research groups such as the use of fluorescence-guided surgery or piezoelectric surgery which have shown promising results in terms of complete mucosal healing [125,126]. During surgical debridement, a fluorescence lamp can be used intraoperatively as a guide for delimiting resection margins of necrotic bone. The most often used light device was the VELScope^®®^ system [127]. According to a recent systematic review, further prospective studies with larger samples are still required to ascertain its clinical validity [128].

**Figure 2:**
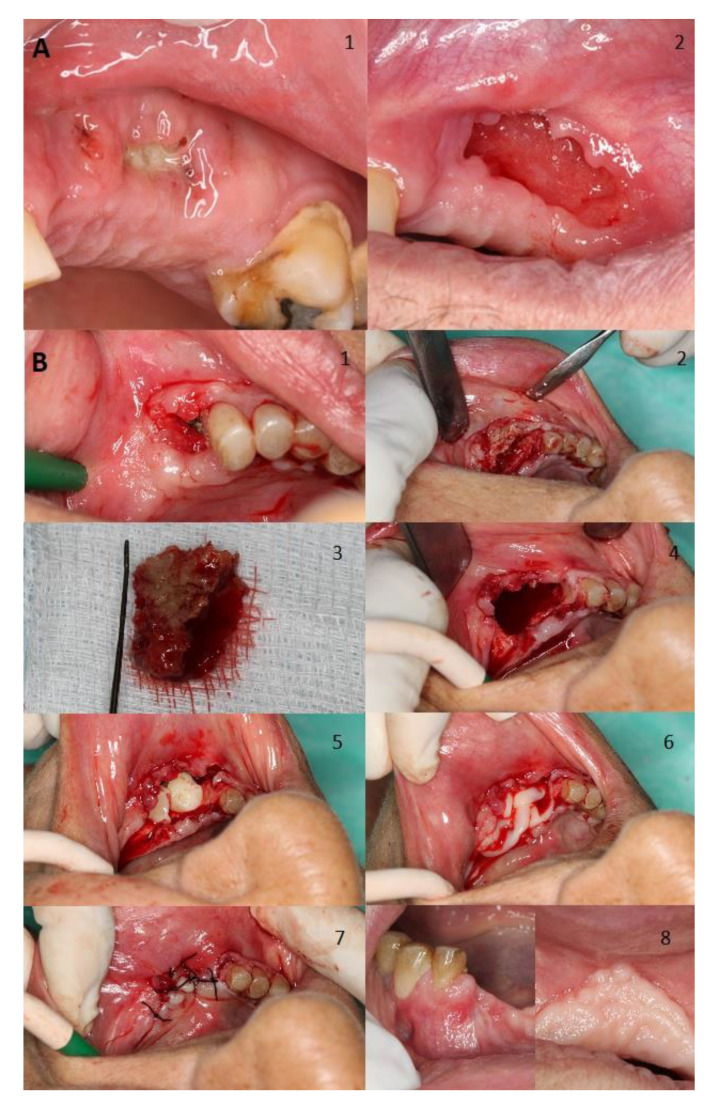
(**A**) Man aged 77 years with prostate cancer without symptomatology developed stage I MRONJ in his left maxilla with bone exposure after receiving zoledronate for suspected bone metastases. Image 1 relates to initial presentation, whilst the second one shows the outcome of treatment with antibiotics and chlorhexidine treatment for 2 months; bone gradually sequestered over time and the soft tissue as the soft tissue eventually closed. (**B**) Woman aged 81 years with osteoporosis who developed stage II MRONJ with bone exposure in her left mandible with severe pain. The patient was initially treated with risedronate but was eventually switched to denosumab. Triggers of necrosis were mainly severe chronic periodontal disease and a dental extraction (Image 1). Initially we performed an incision and flap elevation to visualize affected area (Image 2). Then resection of necrotic bone was performed (Images 3 and 4). Then, an application of L-PRF by Choukroun’s method was performed and primary wound closure was achieved (Image 5, 6, and 7). A month after treatment total resolution was achieved (Image 8).

**Figure 3:**
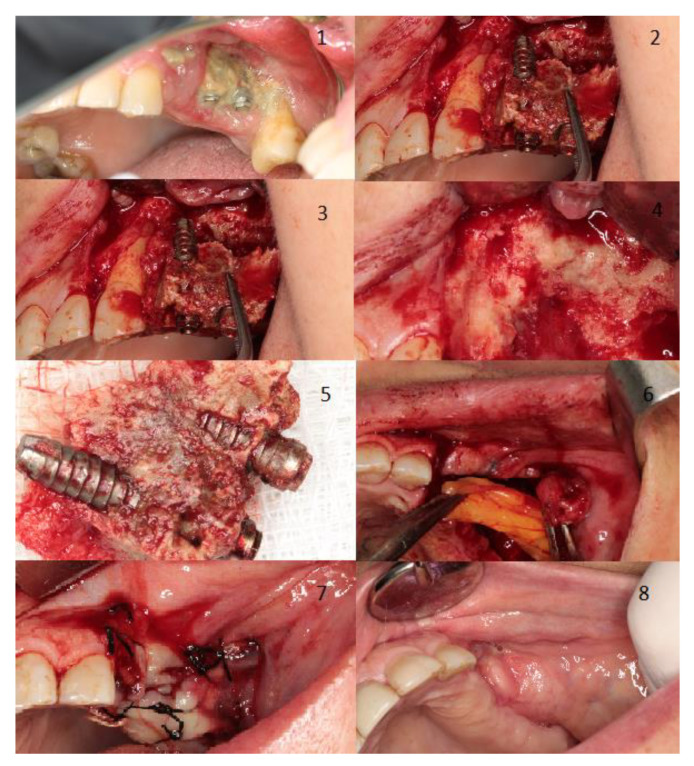
Woman aged 81 years with osteoporosis treat with alendronate. The patient developed stage 3 MRONJ with large areas of bone exposure and pus exudation on the left maxilla plus a chronic oroantral fistula on the left maxillary sinus. The patient experienced severe pain and had severe signs of infection. The trigger factor was dental implants placement (Image 1). Initially, we performed an incision and flap elevation to visualize the affected area (Image 2). Then, two extractions were performed due to the poor dental prognosis, implant extraction, and resection of necrotic bone was also performed (Images 3–5). To close the oroantral fistula, an intraoral bichectomy was performed to harvest tissue. Obtained tissue was placed in the resection area and primary wound closure was achieved (Images 6 and 7). Two months after treatment, total resolution was achieved (Image 8).

Figure 2 shows two examples of management for a spontaneous stage I case, where also shows the treatment for a stage II MRONJ case related to a dental extraction both cases treated in our facilities. Figure 3 displays the management of a stage III case related to dental implants placement.

On the other hand, adjunctive or alternative options for conservative and more radical options have also been proposed such as α-tocopherol, pentoxifylline, ozone therapy, hyperbaric oxygen treatment, or the use of laser therapy with Er:YAG or low light laser therapy [129,130]. Systemic administration of teriparatide with or without a local delivery of recombinant human bone morphogenic growth factor 2 has also shown promising results [131,132]. It is important to emphasize that most of this body of literature relies on case series, retrospective or prospective case-control studies, and a reduced number of clinical trials with a limited number of cases. This fact, added to the diversity of the populations under investigation, specific at-risk drug therapy, and the variety of definitions of successful outcomes, creates a major obstacle to drawing insightful evidence.

## 10. Conclusions

The present review shows relevant shortcomings in almost all MRONJ-related issues critically discussed. Further basic, observational, and interventional studies are necessary to comprehensively understand this complex adverse drug reaction in the orofacial region. Bearing in mind the classical aphorism “there are no diseases, there are ill people”. An individualized evaluation of the patient’s general health and a thorough investigation of the underlying local risk factors are essential, since its underestimation might contribute not only to the onset or lack of response to treatment for MRONJ but also, and more importantly, affect the underlying disease that motivated the prescription of these drugs.

## Data Availability

Data available on request due to restrictions e.g., privacy or ethical.

## Abbreviations

AAOMS	American association of oral and maxillofacial surgeons;
APC	Autologous platelet concentrate;
ASBMR	American society for bone and mineral research;
BP	Bisphosphonate;
BRONJ	Biphosphonate-related osteonecrosis of the jaw;
CI	Confidential interval;
CT	Computed tomography;
CTX	Collagen type I c-telopeptide;
Er:YAG	Erbium YAG;
IDT	Invasive dental treatment;
MMP9	Matrix metallopeptidase 9;
MRI	Magnetic resonance imaging;
MRONJ	Medication-related osteonecrosis of the jaw;
mTOR	Mammalian Target of Rapamycin;
PD	Periodontal disease;
RANKL	Receptor activator for nuclear factor κB ligand;
VEGF	Vascular endothelial growth factor.
